# The roles of glycolysis in osteosarcoma

**DOI:** 10.3389/fphar.2022.950886

**Published:** 2022-08-17

**Authors:** Zuxi Feng, Yanghuan Ou, Liang Hao

**Affiliations:** Departments of Orthopedics, Second Affiliated Hospital of Nanchang University, Nanchang, China

**Keywords:** osteosarcoma, glycolysis, signaling pathway, Cancer Progression, key enzymes

## Abstract

Metabolic reprogramming is of great significance in the progression of various cancers and is critical for cancer progression, diagnosis, and treatment. Cellular metabolic pathways mainly include glycolysis, fat metabolism, glutamine decomposition, and oxidative phosphorylation. In cancer cells, reprogramming metabolic pathways is used to meet the massive energy requirement for tumorigenesis and development. Metabolisms are also altered in malignant osteosarcoma (OS) cells. Among reprogrammed metabolisms, alterations in aerobic glycolysis are key to the massive biosynthesis and energy demands of OS cells to sustain their growth and metastasis. Numerous studies have demonstrated that compared to normal cells, glycolysis in OS cells under aerobic conditions is substantially enhanced to promote malignant behaviors such as proliferation, invasion, metastasis, and drug resistance of OS. Glycolysis in OS is closely related to various oncogenes and tumor suppressor genes, and numerous signaling pathways have been reported to be involved in the regulation of glycolysis. In recent years, a vast number of inhibitors and natural products have been discovered to inhibit OS progression by targeting glycolysis-related proteins. These potential inhibitors and natural products may be ideal candidates for the treatment of osteosarcoma following hundreds of preclinical and clinical trials. In this article, we explore key pathways, glycolysis enzymes, non-coding RNAs, inhibitors, and natural products regulating aerobic glycolysis in OS cells to gain a deeper understanding of the relationship between glycolysis and the progression of OS and discover novel therapeutic approaches targeting glycolytic metabolism in OS.

## 1 Introduction

OS is a malignant bone tumor that primarily affects children and teenagers. It has a low incidence in the general population but is fatal (Davis et al., 2019; Ye et al., 2022). OS occurs in the metaphysis of long bones: the distal femur and proximal tibia (Saraf et al., 2018). One of the important features of OS is its heterogeneity. The complexity of the somatic genome of OS is the main cause of intratumor heterogeneity (Corre et al., 2020). Several genes (TP53, RB, MDM2, ATRX, and DLG2) have been found in OS repeated mutation (Chen et al., 2014). OS has a high proclivity for lung metastasis, contributing to its poor treatment and prognosis (Longhi et al., 2006). OS aggressiveness correlates with osteolysis makers, tumor-infiltrating osteoclasts are frequently found in OS, serum TRACP 5b and MMP9 levels are elevated (Avnet et al., 2008). Over the past few decades, the treatment options for OS have been continuously optimized, and the survival rate of OS patients has improved, but the mortality rate remains high. This necessitates a more comprehensive understanding of OS to identify the best treatment plan (Berner et al., 2015). Few cancer cells grow slowly, while osteosarcoma cells grow very fast and must synthesize large amounts of nutrients to survive.

For rapid growth, cancer cells enhance their metabolism by “metabolic reprogramming.” One of the most common metabolic changes is enhanced glycolysis, also known as the “Warburg effect,” manifested by increased glucose uptake and lactate production (Hsu and Sabatini, 2008). The Warburg effect was first described in the 1920s (Koppenol et al., 2011). Normal cells obtain energy by breaking down glucose through oxidative phosphorylation in the mitochondria. In contrast, cancer cells obtain energy by converting glucose into lactate *via* enhanced glycolysis, either in oxygen or hypoxia (Warburg, 1956). Glycolysis was previously thought to be typically characterized by increased glucose uptake and lactate production but inefficient adenosine triphosphate (ATP) production. This energy source accounts for more than half of the ATP supply of hypermetabolic tumor cells. Recent studies have found that tumor growth *in vivo* is related to the ratio of NAD+/NADH, and reducing the ratio of NAD+/NADH can inhibit cell proliferation (Gui et al., 2016).

When the demand for NAD exceeds the demand for ATP, cells undergo aerobic glycolysis (Luengo et al., 2021). Studies have shown that enhanced glycolysis is critical for cancer cells’ rapid growth and metastasis (Kroemer and Pouyssegur, 2008). Reliable and effective therapeutic drugs can be explored and developed by targeting the glycolytic pathway (Dang et al., 2009; Li et al., 2018). Over the past few decades, glycolysis has been extensively demonstrated for the growth, invasion, and treatment of various tumors, including OS. However, there is no systematic study and discussion on how glycolysis regulates and affects the progression and treatment of OS. This study summarizes the roles of key glycolysis enzymes, signaling pathways, non-coding RNAs, inhibitors, and natural products in OS and explores metabolic-targeted treatment strategies for OS.

## 2 The glycolysis in osteosarcoma

OS drugs, doxorubicin (ADR) or paclitaxel in combination with the glycolysis inhibitor 2-DG, are significantly more effective in reducing tumor growth than either drug alone (Maschek et al., 2004). This indicates that the glycolysis inhibitors combined with chemotherapeutic drugs improve the therapeutic effect in OS, so it is necessary to understand the mechanism of glycolysis in OS deeply. Designing small-molecule inhibitors target some key enzymes in the glycolytic pathway is a reliable solution for studying OS therapeutic strategies. For example, targeting glycolytic regulator (M2-PK) by synthetic peptide aptamers reduces the proliferation ability of OS cells (Spoden et al., 2008). According to many studies, enhanced glycolysis is the main metabolic alteration in OS, while many glycolytic enzymes have been shown to promote the tumorigenic activity of OS cells and are associated with poor prognosis in patients (Sottnik et al., 2011; Zhuo et al., 2015; Shen et al., 2019). Furthermore, this study reviewed some key glycolytic enzymes, signaling pathways, transcription factors, and non-coding RNAs in the glycolytic in OS cells.

### 2.1 Key enzymes of glycolysis in osteosarcoma

**FIGURE 1:**
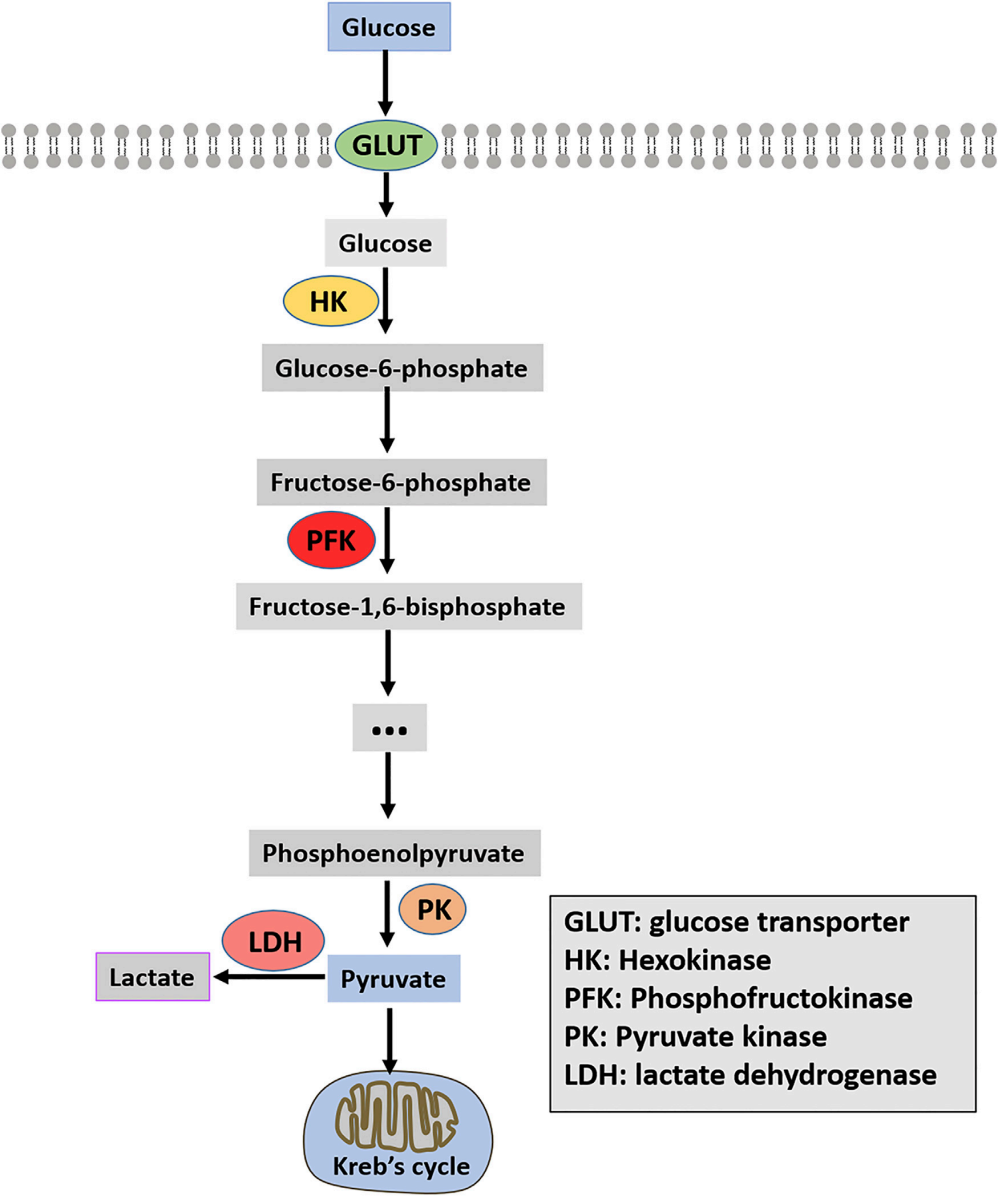
Schematic diagram of glycolysis.

Enhancing glycolysis in cancer tissues and cells is closely related to the overexpression or enhanced activity of key glycolysis enzymes (Bian et al., 2022). Glycolysis-related enzymes include three rate-limiting enzymes: hexokinase (HK), phosphofructokinase (PFK), and pyruvate kinase (PK). Moreover, glucose transporter (GLUT) and lactate dehydrogenase (LDH) are also summarized in this paper and shown in Figure 1.

#### 2.1.1 HK2

##### 2.1.1.1 The function of HK2

HK2 is one of the subtypes of hexokinase, which functions as the first rate-limiting glycolysis enzyme in the glycolysis pathway and converts glucose to glucose-6-phosphate (G6P) (Wilson, 2003). HK2 is the most efficient of all subtypes of HK, and it has both an N-terminal active site domain and a C-terminal active site domain (Xu and Herschman, 2019). Glucose quickly enters the cell through the glucose transporter, where HK2 binds ATP and intracellular glucose to efficiently catalyze the production of glucose-6-phosphate (G6P), which promotes glycolysis (Mathupala et al., 2006). HK2 is also related to the progression of several cancers. HK2 is abnormally expressed in high amounts in many cancers, which is very important for tumor initiation, and maintenance and is closely related to cancer treatment and prognosis (Gong et al., 2012; Patra et al., 2013; Sato-Tadano et al., 2013).

##### 2.1.1.2 The roles of HK2 in osteosarcoma

Significantly, HK2 is overexpressed in OS and is closely related to Ki67. Ki-67, also known as MKI-67, is a protein encoded by the MKI67 gene in humans. Expressed only in actively proliferating cells, is a proliferation-associated nuclear antigen. Clinically, ki67 is mainly used to label cells in the proliferative cycle and due to its overexpression in cancer cells, Ki-67 has been proposed as a prognostic biomarker for cancer (Li et al., 2015). The 5-year survival rate of patients with ki67 expression in osteosarcoma patients is extremely low (Zeng et al., 2021). The knockdown of HK2 causes a decrease in aerobic glycolysis, increasing cell apoptosis and decreasing cell colony formation ability. Furthermore, the effects mentioned above of HK2 can be blocked by the glycolysis inhibitor, 2-deoxy-d-glucose (2-DG), which further indicates that the effect of HK2 on the growth of OS is achieved by regulating the glycolysis pathway (Zhuo et al., 2015). Besides, HK2 is the direct transcriptional target of the classical signal pathway nuclear factor kappa B (NF-κB). HK2 overexpression can restore metabolic transfer induced by inhibiting NF-κB activity in OS cells and enhance the glycolysis pathway (Londhe et al., 2018). HK2 functions as a target for microRNAs (miRNAs) in OS cells (Song et al., 2017; Sun L. et al., 2020; Wan et al., 2021). Liu C. et al. (2019) suggested that low expression of miR-185 in OS negatively regulates the expression of HK2. Overexpression of miR-185 significantly suppressed the proliferation and migration of OS cells. However, HK2 overexpression could significantly weaken the inhibitory effect of miR-185 on glycolysis in OS cells. By inhibiting HK2, miR-185 inhibits glucose metabolism in OS cells, making it an excellent target for drugs. In addition, HK2 can be regulated by the ELK1-miR-134-PTBP1 signaling pathway, which affects the glycolysis and chemical resistance of OS cells (Zhang et al., 2021b). circRNA can also affect the progress of glycolysis and OS by regulating the expression of HK2 (Gu et al., 2020). Interestingly, HK2 can also be regulated by lncRNA. LncRNA-SARCC can increase the sensitivity of OS cells to cisplatin. The mechanism may be that miR-143 inhibits the expression of HK2 and reduces the glycolysis level of OS cells. The mechanism of lncRNA-SARCC-miR-143-HK2 provides a new therapeutic approach for studying cisplatin resistance in OS (Wen et al., 2020). A study illustrated that iron increased the expression of HK2 and GLUT1, and regulated the AMPK/mTORC1 signaling pathway (Ni et al., 2020). Overall, these results indicate that targeting the HK2 might be a potential therapeutically valuable strategy in OS.

#### 2.1.2 Phosphofructokinase

##### 2.1.2.1 The function of phosphofructokinase

The primary function of fructose-6-phosphate-1-kinase (PFK-1) is to catalyze the conversion of fructose-6-phosphate to fructose-6-diphosphate and adenosine diphosphate (ADP), thereby promoting glycolysis. As this process is irreversible, phosphofructokinase (PFK) becomes the second rate-limiting enzyme in glycolysis (Uyeda, 1979). The catalytic activity of PFK-1 is regulated by some metabolites, small molecule inhibitors, and intracellular proteins, such as adenosine triphosphate (ATP), F-6-P, and 2, 6-diphosphate fructose 6-diphosphate (F-2,6-BP), and adenosine diphosphate (ADP) (Sola-Penna et al., 2010; Bartrons et al., 2018). As a product of catalyzation by 6-phosphofructo-2-kinase/fructose-2,6-bisphosphatase (PFK-2/FBPase-2, PFKFB), F-2,6-BP is one of the regulatory substances and the most potent positive allosteric effector of PFK-1 (Yi et al., 2019). Four different genes (PFKFB1, PFKFB2, PFKFB3, and PFKFB4) encode PFK-2/FBPase-2 (Ros and Schulze, 2013). These four genes are expressed differently in different human tissues and organs. PFKFB1 mainly exists in the liver and skeletal muscle, PFKFB2 is highly expressed in the myocardium, PFKFB3 is generally expressed and PFKFB4 is highly expressed in the testis (Yi et al., 2019). In mammals, three subtypes of PFK have been identified: PFK-M (muscle), PFK-P (plasma), and PFK-L (liver). The homology between PFK-M and PFK-L is about 68.6%, between PFK-M and PFK-P is about 70.3%, and between PFL-L and PFL-P is about 66.6% (Sola-Penna et al., 2010). The effect of PFK on tumor cells has been extensively researched. PFK is regulated by carcinogenic proteins (HIF-1α and c-myc) or tumor suppressor protein (p53) in many tumors, which affects tumor progression by altering the rate of glycolysis (Moreno-Sanchez et al., 2007; Yalcin et al., 2009).

##### 2.1.2.2 The roles of phosphofructokinase in osteosarcoma

The role of PFKFB1 and PFKFB4 in OS has not been reported. PFKFB1 has four splice variants: PFKFB1-201, PFKFB1-202, PFKFB1-203 and PFKFB1-204 (Lee et al., 2003; Ros and Schulze, 2013). PFKFB-1 is lowly expressed in normal cells, but overexpressed in cancer cells (Minchenko et al., 2003; Kotowski et al., 2021). For example, the expression of PFKFB-1 is significantly correlated with the prognosis of bladder cancer patients (Zhang C. et al., 2020). PFKFB4 is overexpressed in multiple human cancers such as lung adenocarcinoma, supports tumor growth by synthesizing F-2,6-BP and is required for glycolysis in response to hypoxia and tumor growth (Chesney et al., 2014). Studies have demonstrated that PFKFB2, as the substrate of tumor inhibitor miR-1297, is negatively regulated by miR-1297 and affects the proliferation of OS by regulating glycolysis (Pan et al., 2020). SRC/ERK/C-MYC/PFKFB2 signal pathway in OS cells affects the glycolysis pathway by regulating PFKFB2 expression, affecting its proliferation (Zhao et al., 2018). Moreover, the tumor suppressor gene miR-26b is down-regulated in OS. It regulates the expression of the glycolysis-related protein (LDHA, GLUT1), invasion, and cell cycle markers (such as MMP-9, MMP-2, cyclin D1, and p27) by inhibiting the expression of mRNA and protein of PFKFB3, resulting in malignant progression of OS (Du et al., 2015). Recent studies have revealed that Rho-related coiled-coil kinase (ROCK) includes two members: ROCK1 and ROCK2. Dysregulated expression of ROCK is closely associated with enhanced metastatic ability and reduced patient survival in various cancers (de Sousa et al., 2020). Both ROCK2 and PFKFB3 proteins have significantly high expression in OS. Regarding mechanism, ROCK2 stabilizes PFKFB3 expression by blocking its ubiquitination degradation. Low-level ROCK2 knockouts can save OS cells from proliferation and metastatic loss by upregulating PFKFB3 (Deng et al., 2019). The ubiquitin-like protein FAT10 is highly expressed in various cancers (colorectal, liver, and gastric) and promotes cancer progression (Aichem and Groettrup, 2016). Similarly, ubiquitin-like protein FAT10 is highly expressed in OS tissues, while high levels of FAT10 are closely associated with tumor growth and survival time of OS patients. Experimental results showed that FAT10 upregulates the protein expression level of PFKFB3 by directly binding to EGFR and inhibiting its ubiquitination and degradation, thus inducing glycolysis (Deng et al., 2020). It can be observed that PFK, as a glycolytic enzyme, also affects malignant behaviors of OS, such as cell growth and metastasis.

#### 2.1.3 Pyruvate kinase

##### 2.1.3.1 The function of pyruvate kinase

Pyruvate kinase (PK) is the last rate-limiting enzyme in the glycolysis pathway. Its function is to catalyze the conversion of phosphoenolpyruvate (PEP) to pyruvate and convert ADP to ATP, thus promoting glycolysis (Harris et al., 2012). PK is encoded by two genes, PKLR and PKM, and it is divided into four subtypes: PKL (liver type competition), PKR (red blood cell competition), PKM1 (muscle competition isozyme M1), and PKM2 (muscle competition isozyme M1) (Dayton et al., 2016). The distribution of these four subtypes in the human body is diverse. PKL subtypes are highly expressed in kidney, liver and red blood cells; PKR mainly exists in red blood cells; PKM1 is enriched in the myocardium, skeletal muscle and brain tissues; PKM2 is mainly distributed in the brain and liver tissues (Israelsen et al., 2013). PKM2 is the most powerful and widely studied subtype and has abnormally high expression in many cancers, such as colorectal tumors, breast tumors, lung tumors, prostate tumors, and ovarian tumors, while the expression levels in the pharynx and testicular tumors are below or close to the critical value (Munoz-Colmenero et al., 2015). PKM2 can regulate the activity and localization of proteins through post-transcriptional modifications such as phosphorylation and acetylation, thus regulating cell proliferation ability (Israelsen and Vander Heiden, 2015). Lv et al. (2013) reported that the carcinogenic form of fibroblast growth factor receptor type 1 could directly phosphorylate PKM2 tyrosine residue 105 (Y105) to inhibit PKM2 and tumor growth. Furthermore, PKM2 was acetylated by p300 acetyltransferase at the K433 site, which interfered with the binding of FBP to block PKM2 activation. It promoted the nuclear accumulation and kinase activity of PKM2, thereby promoting the proliferation of cancer cells.

##### 2.1.3.2 The roles of Pyruvate kinase in osteosarcoma

Of the four competing subtypes, only PKM2 is more studied in OS. Notably, PKM2 is expressed abnormally high in OS tissues and is more abundant than para cancerous tissues (60.2 vs. 26.1%, *p* < 0.001). In addition, high expression of PKM2 is positively correlated with kennel stage and distant metastasis. The higher the expression level of PKM in OS patients, the higher the overall survival (OS) time (Liu Z. X. et al., 2016). Moreover, circPVT1 binds to miR-423-5p and activates the Wnt5a/Ror2 signal through saponification of miR-423-5p, promoting OS glycolysis (upregulated by HK2, GLUT1, LDHA, and PKM2), proliferation and transfer (Wan et al., 2021). Pu et al. (2022) demonstrated that the interaction between lncRNA colon cancer-associated transcript-1 (lncCCAT1) and PKM2 promotes the phosphorylation of sterol regulatory element binding protein 2 (SREBP2) and enhances the Warburg effect, adipogenesis, and OS cell growth. As a direct target of miR-491-5p, PKM2 was negatively regulated by miR-491-5 in OS cells and PKM2 overexpression could rescue the inhibitory effect of miR-491-5p on the proliferation of OS cells (Chen T. et al., 2018). The knockdown of PKM in OS cells decreased the expression levels of cyclin D1 and Bcl-2 and increased the expression levels of Bax, caspase-3, and PARP. Moreover, knockdown of PKM inhibited the growth of OS cells and induced G1 cell cycle arrest and apoptosis in OS cells. In addition, the knockdown of PKM2 significantly inhibited the metastatic ability of OS cells. *In vivo* experiments displayed that the knockdown of PKM2 can reduce the growth rate of OS and induce the structural deterioration of tumor tissue (Yuan et al., 2018). Glucagon-like peptide 2 (GLP-2), a glucagon precursor-derived peptide, promotes the release of ingested nutrients from enteroendocrine cells (Suzuki et al., 2020). GLP2 inhibits the proliferation of OS cells both *in vivo* and *in vitro*. The specific mechanism is that GLP2 inhibits the expression of NF-κB expression in OS cells, resulting in decreased PKM2 and CyclinD1 (Lu et al., 2018). According to a case report, miR-1294 is down-expressed in OS cells. Overexpression of miR-1294 inhibits the growth and metastasis of OS cells, and induces G0/G1 stagnation and apoptosis. Besides, PKM2 is a direct target of miR-1294 in OS cells, PKM2 overexpression saves the decline of cell proliferation, migration, and invasion caused by miR-1294 overexpression (Yuan et al., 2020). IRF7 is a transcription factor and one of the interferon regulatory factors (IRFs). IRFs play important roles in cell differentiation, apoptosis, and cell cycle regulation in tumor development (Chen et al., 2017). PKM2 mediates the effect of IRF7 on glycolysis in OS cells. IRF7 inhibits PKM2 transcription, thus suppressing the glycolysis and resulting in a decline in the proliferation of OS cells (Li et al., 2022). Interestingly, PKM2 is closely related to chemotherapy drug resistance of OS stem cells. PKM2 overexpression can lead to the resistance of cisplatin by OS stem cells. Metformin treatment combined with PKM2 knockdown significantly reduces the semi-maximum inhibitory concentration (IC50) of cisplatin on HOS OS stem cells (Shang et al., 2017).

#### 2.1.4 Glucose transporters

##### 2.1.4.1 The function of glucose transporters

Human GLUTs are a 14-member family encoded by the SLC2 gene (Deng and Yan, 2016). Increased glucose uptake and metabolism are hallmarks of cancer cells, and the primary function of GLUTs is to facilitate glucose uptake across the plasma membrane (Barron et al., 2016). Generally, GLUT1 is expressed in almost all tissues but is abnormally expressed in high amounts in numerous cancers, including lung cancer, breast cancer, hepatocellular carcinoma, and kidney cancer (Barron et al., 2016). GLUT2 mainly transports glucose in hepatocytes and has a very high K_m_ value and a high affinity for glucosamine (Deng and Yan, 2016). Several studies have shown that the high affinity and transportability of GLUT3 to glucose are prominent in neurons, and the expression of GLUT3 in gliomas and astrocytoma is significantly higher than that of GLUT1 (Barron et al., 2016). GLUT4 is mainly distributed in adipocytes, cardiomyocytes, and skeletal muscle cells *in vivo* and is essential for regulating systemic glucose homeostasis (Mueckler and Thorens, 2013).

##### 2.1.4.2 The roles of glucose transporters in osteosarcoma

Numerous studies have reported that GLUT1 is a significant independent prognostic factor for many tumors, including OS. Kubo et al. (2015) immunohistochemically determined the expression of GLUT1 and the angiogenic activity of CD34 positive microvessels in patients with high-grade OS. Studies have suggested that glucose metabolism might negatively affect angiogenesis. GLUT1 is a potential marker of survival and drug therapy in patients with OS, with important implications for the prognosis of OS. GLUT1, as the downstream target gene of HIF1a, often affects the growth of OS and is the key protein related to angiogenesis and energy metabolism (Nepal et al., 2011). In addition, the protein level and mRNA level of GLUT1 are abnormally overexpressed in OS, and high levels of GLUT1 are significantly associated with lymph node metastasis, age and low survival rate (Fan et al., 2017). Knockdown of GLUT1 significantly slowed the proliferation of OS cells *in vitro* and *in vivo* (Fan et al., 2010; Jian et al., 2015). A study suggested that GLUT1 and GLUT4, downstream substrates of tumor suppressor p53, were negatively regulated by p53 protein to reduce glucose transport. The inhibitory effect of P53 on the transcriptional activity of the GLUT4 promoter was significantly greater than that on GLUT1 promoter. thereby degrading metabolic intensity and inhibiting tumor growth (Schwartzenberg-Bar-Yoseph et al., 2004). The main function of GLUT1 is to transport glucose, which is no different in OS. Studies have reported that GLUT1 is regulated by insulin in MG-63 cells of OS. Although insulin does not affect the expression of GLUT1, it can induce translocation to the plasma membrane (Cifuentes et al., 2011). Remarkably, GLUT1 can also be regulated by lncRNA. The expression level of HAND-AS1 was lower in OS than in normal tissues. Knockdown of HAND-AS1 promotes GLUT1 protein expression, suppressing OS cell proliferation. Non-coding RNA of HAND2-AS1 targets glucose metabolism and inhibits the proliferation of cancer cells in OS (Chen S. et al., 2019). In addition, miR-328-3p inhibited GLUT1 expression and glucose uptake rate in OS cells, and hBERA/miR-328 combined with OS chemotherapeutic drugs cisplatin or doxorubicin could significantly inhibit the proliferation of OS cells (Yi et al., 2020). OS is a highly invasive tumor, which is an important reason for its poor prognosis. As a tumor biological function regulator, LAIR-1 can inhibit the expression of GLUT1, thus reducing the expression of EMT-related molecules in OS cells and inhibiting the metastatic ability of OS cells (Zhang J. et al., 2020). Carcinogen STC2 enhances glycolysis and promotes the growth of OS cells by upregulating the expression of GLUT1 (Yu et al., 2021). The role of GLUT2 in OS has not been reported so far. A study demonstrated that ELK1 promotes the expression of GLUT3, HK2, PDK1 and PTBP1 by inhibiting miR-134 expression, while enhancing the chemical tolerance and aerobic glycolysis of OS cells (Zhang et al., 2021b). Altogether, glucose transporters (GLUTs), especially GLUT1, play a critical role in the glycolytic pathway and is a putative candidate target in OS treatment.

#### 2.1.5 LDHA

##### 2.1.5.1 The function of LDHA

Lactate dehydrogenase (LDH) catalyzes pyruvate to lactate and is a vital oxidoreductase in the glycolytic pathway. LDH comprises two subunits, LDH-A and LDH-B, assembled into five different isozymes (LDH1, LDH2, LDH3, LDH4, and LDH5). LDH-A, encoded by the LDHA gene, is the main skeletal muscle form and has a high affinity for pyruvate. LDH-B is encoded by the LDHB gene and exists primarily in the myocardium (Dawson et al., 1964; Ding et al., 2017). Due to the high affinity for pyruvate, LDHA prioritizes the conversion of pyruvate to lactic acid and NADH to NAD+. Simultaneously, LDHB has a high affinity for lactic acid under anaerobic conditions. When oxygen is sufficient, priority is given to converting lactic acid to pyruvate and NAD + to NADH (Urbanska and Orzechowski, 2019). Immunohistochemistry experiments indicated that LDHB expression in normal and cancer tissues had no significant difference. However, LDHA was found to be highly expressed in most cancer cells, which is the main reason why LDHA can be used as a biomarker of many malignant tumors, such as gastric cancer (Kolev et al., 2008), testicular germ cell tumor (Augoff and Grabowski, 2004), glioma (Hu S. et al., 2016) and melanoma (Brand et al., 2016). The function of LDHB in tumors has also been reported (Doherty and Cleveland, 2013). LDHB is expressed abnormally high in lung cancer cell lines and is closely associated with the survival rate of patients with lung adenocarcinoma (McCleland et al., 2013). As a key enzyme in the glycolysis pathway, LDHB is also an essential protein for triple-negative breast cancer. LDHB and other related glycolysis enzymes are significantly upregulated in triple-negative breast cancer. Knocking down LDHB suppresses the growth of breast cancer cells (McCleland et al., 2012).

##### 2.1.5.2 The roles of LDHA in osteosarcoma

In OS, glycolytic enzymes such as LDHA, GLUT1, and HK1 can be negatively regulate the phenolic compound apigenin (4′,5,7-trihydroxyflavone), which attenuates the Warburg effect, mediated by PI3K/Akt/mTOR signal pathway dehydrogenase (Shi et al., 2022). In 2019, Wang et al. found three cell-active hLDHA inhibitors (38, 63, and 374) by targeting human lactate, and related experimental evidence that these inhibitors suppressed the proliferation of MG-63 cells. The IC_50_ values were 6.47 μM, 2.93 μM, and 6.10 μM, and hLDHA_50_ values were 3.03 μM, 0.63 μM, and 3.26 μM for inhibition by EC (Wang F. et al., 2020). Other studies have also designed a variety of inhibitors for LDHA and confirmed their inhibitory effect on LDHA by enzymatic assay *in vitro* and strongly inhibited the proliferation of OS cells by cytotoxicity assay (Cui et al., 2016; Fang et al., 2017). MicroRNAs (miRNAs) have been widely studied in OS growth, invasion, and migration. The miR-33b inhibits glycolysis by targeting the key enzyme lactate dehydrogenase A (LDHA), suppressing OS cells’ growth (Zheng et al., 2018). In 2016, compared with normal osteoblasts (hFOB1.19), LDHA was upregulated in OS cell lines. Treatment of OS cells with FX11, an LDHA inhibitor, down-regulated the activity of LDHA and suppressed the growth and metastasis abilities of OS cells in a dose-dependent manner. Moreover, the study also reported that 2-DG, a glycolysis inhibitor, completely blocked cell proliferation and invasion mediated by LDHA, suggesting that the carcinogenic activity of LDHA in OS may be dependent on glycolysis. Interestingly, inhibiting the pharmacological effects of c-myc or HIF-1α reduces the expression of LDHA (Gao et al., 2016). LDHA was also closely related to cisplatin resistance in OS. Bioinformatics analysis and luciferase assay demonstrated that glycolytic enzyme LDHA was the downstream substrate of miR-329-3p in OS cells. LDHA overexpression in miR-329-3p overexpressed cisplatin-resistant cells could restore glucose metabolism and increase cisplatin resistance in OS cells (Li G. et al., 2021). The miR-323a-3p negatively regulated mRNA and protein levels of LDHA in OS cells. The expression levels of LDHA and miR-323a-3p in OS tissues showed a negative correlation. LDHA promotes glycolysis in OS, while miR-323a-3p overexpression reduces the ability of OS cells to produce lactic acid (Chen H. et al., 2018). Circular RNAs (circRNAs) are critical in OS biology. For example, circATRNL1 is highly expressed in OS tissues and cells. CircATRNL1 regulates the expression of LDHA, promoting glycolysis and the progression of OS (Zhang et al., 2021a). Furthermore, LDHA is regulated by various circRNAs, which mediates glycolysis and the growth of OS (Wan et al., 2021; Jiang X. et al., 2022). A new study stated that abnormally high expression of KDM6B in OS promotes tumor metastasis in OS by positively regulating the expression level of LDHA, which raises the prospect of OS (Jiang Y. et al., 2021). There are also a few studies on the role of LDHB in OS. Remarkably, LDHB was highly expressed in OS cells, and the mRNA expression level of LDHB was significantly higher in metastatic OS. Furthermore, the poor prognosis of patients with OS frequently correlated with a high expression of LDHB. LDHB knockdown inhibits the growth and metastasis of OS cells. Statistical analysis of clinicopathological characteristics of LDHB disclosed that the expression levels of LDHB were related to the TNM stage of the tumor, recurrence, and survival in OS patients. Therefore, LDHB can be regarded as a prognostic marker of cancer recurrence and poor overall survival of OS (Li et al., 2016). Similarly, LDHB is highly expressed in OS tissues and cells, and the existence of the miR-141-3p/FUS/LDHB axis enhances the growth, migration, and invasion of OS cells (Wang, 2020).

### 2.2 Potential natural products of glycolysis in osteosarcoma

Many natural compounds and synthetic reagents targeting glycolytic enzymes have been developed, with related cell experiments and clinical trials proving that they can inhibit OS progression. For example, Icariside II, an active flavonoid extracted from *Epimedium koreanum* *Nakai* (Berberidaceae), has anticancer effects in various cancer cells (Kang et al., 2012; Wu et al., 2015) (Geng et al., 2014). It is a natural mTOR inhibitor that inhibits aberrant glycolysis in OS by inhibiting the cap-dependent translation of c-myc through mTORC1-4E-BP1 axis (Zhang et al., 2016). Bavachinin is a flavonate from the seeds of *Proralea corylifolia L.*, native to eastern Asia, inducing HIF-α ubiquitin and proteasome-mediated degradation and inhibiting the synthesis of HIF-1 and the glucose metabolism of OS (Nepal et al., 2012). Resveratrol is a natural polyphenol extracted from grapes, mulberries, peanuts, and other plants. In OS, resveratrol upregulates Cx43 and E-cadherin and inhibits the Wnt/β-catenin signaling pathway, thereby inhibiting OS proliferation and glycolysis, inducing apoptosis, and reducing the invasiveness of U2-OS cells *in vitro* (Xie et al., 2017). Quercetin, an all-natural chemical found in a variety of plants and foods (Harborne and Williams, 2000), induces apoptosis in osteosarcoma cells through mitochondrial dysfunction and Akt dephosphorylation (Xie et al., 2011). The isoflavones formononetin and calycosin are the two main active components of traditional Chinese medicine Radix astragali. They are involved in the inactivation of ERK and Akt pathways, thereby inducing apoptosis of OS cells, and formononetin is more effective than calycosin (Yang et al., 2014). Oxymatrine and matrine are alkaloids extracted from the roots of *Sophora flavescens* and have various pharmacological activities. Oxymatrine induces human bone by inhibiting PI3K/Akt pathway mitochondrial-dependent apoptosis in sarcoma cells (Wei et al., 2014; Zhang et al., 2014). *Selaginella tamariscina* is a traditional medicinal plant for treating advanced cancer in the East and exerts an anti-metastatic effect on OS cells by inhibiting p38 and Akt signaling pathways (Yang et al., 2013). Tanshinone I is isolated from the Chinese herbal medicine *Salvia miltiorrhiza* and can significantly downregulate circ_0000376 expression in OS cells and inhibit the viability, migration, and invasion of OS cells through circ_0000376/miR-432-5p/BCL2 axis, glycolysis and trigger apoptosis of OS cells (Ye et al., 2022). Punicalagin is a polyphenolic compound extracted from pomegranate, which downregulates the expression of p65, survivin, XIAP, CIAP2 and other proteins, and inhibits the proliferation and metastasis of OS cells by inhibiting the NF-κB signaling pathway (Wang X. Z. et al., 2020). Chlorogenic acid (CGA) is a phenolic compound contained in plant-related products, although related levels are also found in other plant-related products such as herbs, fruits, and vegetables. Green coffee beans are the main source of CGA (Liang and Kitts, 2015). which activates ERK1/2 and inhibits the proliferation of OS cells (Sapio et al., 2020). Oridonin, a diterpenoid extracted from the herb Rubescens, inhibits the proliferation, migration, and invasion of OS cells by inhibiting the expression of matrix metalloproteinases and STAT3 signaling pathway (Du et al., 2019). Amentoflavone, a flavonoid extracted from *Selaginella tamariscina*, inhibits OS progression by inhibiting ERK/NF-κB signaling pathway (Lee et al., 2019). Oleanolic acid (OA), a naturally occurring triterpenoid, induces apoptosis in OS cells by inhibiting Notch signaling while inhibiting the proliferation and invasion of OS cells by suppressing SOX9/Wnt1 signaling (Xu et al., 2018) (Chen X. et al., 2021). The above potential natural products of glycolysis in osteosarcoma are displayed in Tables 1, 2.

**TABLE 1:** List of glycolysis inhibitors that target OS cells.

Inhibitors	Target glycolysis enzymes	References
Cytochalasin B	GLUT	Ogawa et al. (2021)
STF-31	GLUT	Kraus et al. (2018)
GLU-MTX (glucose-methotrexate conjugate)	GLUT1	Wozniak et al. (2021)
Insulin and parathyroid hormone (PTH)	GLUT1	Thomas et al. (1995)
KRH102053	HIF-1α	Nepal et al. (2011)
NSC-134754 and NSC-643735	HIF-1α	Chau et al. (2005)
Bavachinin	HIF-1α	Nepal et al. (2012)
Icariside II	HIF-1α	Choi et al. (2008)
FX11	LDHA	Gao et al. (2016)
Compound 11	LDHA	Fang et al. (2017)
2-((3-cyanopyridin-2-yl) thio) acetamide-containing compounds	LDHA	Cui et al. (2016)
Metformin	PKM2	Shang et al. (2017)
63	LDHA	Fei Wang et al. (2020)
12	PDK2	Cao et al. (2019)

**TABLE 2:** List of potential small molecule inhibitors and drugs for OS treatment in basic experimental or clinical trials.

Inhibitors	Target signaling pathway	References	Inhibitors	Target signaling pathway	References
LY294002	Akt	Li et al. (2019)	Temsirolimus	mTOR	Bagatell et al. (2014)
Miltefosine	Akt	Pan et al. (2019)	RES-529	mTOR	Hu et al. (2018)
MK-2206	Akt	Kuijjer et al. (2014)	XL388	mTOR	Zhu et al. (2016)
Perifosine	Akt	Yao et al. (2013)	11-7082	NF-κB	Yan et al. (2015)
S473D	Akt	Sun et al. (2022)	BAY11-7082	NF-κB	Liang et al. (2012)
Curcumin	Akt	Peng et al. (2014)	DHMEQ	NF-κB	Castro-Gamero et al. (2012)
Formononetin	Akt	Liu et al. (2014b)	PDTC	NF-κB	Hongxu Zhang et al. (2017b)
Quercetin	Akt	Xie et al. (2011)	BKM120	PI3K	Bo Liu et al. (2014)
A-674563	Akt	Dai et al. (2017)	BYL719	PI3K	Gobin et al. (2015)
CCT128930	Akt	Sun et al. (2020)	NVP-BKM120	PI3K	Bavelloni et al. (2019)
JQ-1	c-myc	Jiang et al. (2021)	Wortmannin	PI3K	Lee et al. (2013)
Cediranib	EGFR	van Cruijsen et al. (2010)	PI-103	PI3K	Adamski et al. (2013)
Erlotinib	EGFR	Liu et al. (2020)	ZSTK474	PI3K	Jinqiong Jiang et al. (2021)
Gefitinib	EGFR	Wang et al. (2021)	Oxymatrine	PI3K/Akt	Zhang et al. (2014)
Lapatinib	EGFR	Maloney et al. (2020)	Selaginella	PI3K/Akt	Yang et al. (2013)
ZD6474	EGFR	Liu et al. (2015)	Icariside II	PI3K/Akt/mTOR	Geng et al. (2014)
CZ415	mTOR	Yin et al. (2017)	L740Y-P/LY294002	PI3K/Akt	Yan Huang et al. (2020)
PP242	mTOR	Wang et al. (2014)	NVP-BEZ235	PI3K/mTOR	Manara et al. (2010)
RAD001	mTOR	Moriceau et al. (2010)	VS5584	PI3K/mTOR	Jing-Yi Sun et al. (2020)
Ridaforolimus	mTOR	Chawla et al. (2012)	LY2109761	TGF-β	Ren et al. (2015)
Everolimus	mTOR	Pignochino et al. (2013)	A005	PI3K/mTOR	Lian et al. (2019)
Rapamycin	mTOR	Xie et al. (2013)	HS-173	PI3K	Ailiang Zhang et al. (2017)
Punicalagin	NF-κB	Wang et al. (2020)	Sorafenib	NF-κB	Ching-Hsuan Wu et al. (2021)
Bmi-1	NF-κB	Liu et al. (2017)	Tegavivint	Wnt/β-catenin	Nomura et al. (2019)
Curcumin	Wnt/β-catenin	Leow et al. (2010)	Tideglusib	GSK-3β/NOTCH1	Wei et al. (2021)
WT161	AKT	Sun et al. (2021)	Chlorogenic acid	ERK1/2	Sapio et al. (2020)
Polyphyllin VII	JNK	Li et al. (2021)	Oridonin	TGF-β1/Smad2/3	Sun et al. (2018)
RepSox	JNK/Smad3	He et al. (2021)	IWR-1	Wnt/β-catenin	Martins-Neves et al. (2018)
Dickkopf 3	Wnt/β-catenin	Hoang et al. (2004)	Embelin	PI3K/Akt	Qian et al. (2018)
Cobimetinib	MEK/ERK	Ma et al. (2020)	Amentoflavone	ERK/NF-κB	Pan et al. (2017)
RES-529	mTORC1/2	Hu et al. (2018)	INK-128	mTORC1/2	Jiang and Zeng, (2015)
Caffeine	AKT/mTOR/S6K	Miwa et al. (2012)	Arsenic trioxide	MAPK	Tingting et al. (2010)
Oleanolic acid	mTOR	Zhou et al. (2011)	Epigallocatechin-3-gallate (EGCG)	MEK/ERK	Tang et al. (2015)

### 2.3 Signaling pathways linking glycolysis and osteosarcoma

**FIGURE2:**
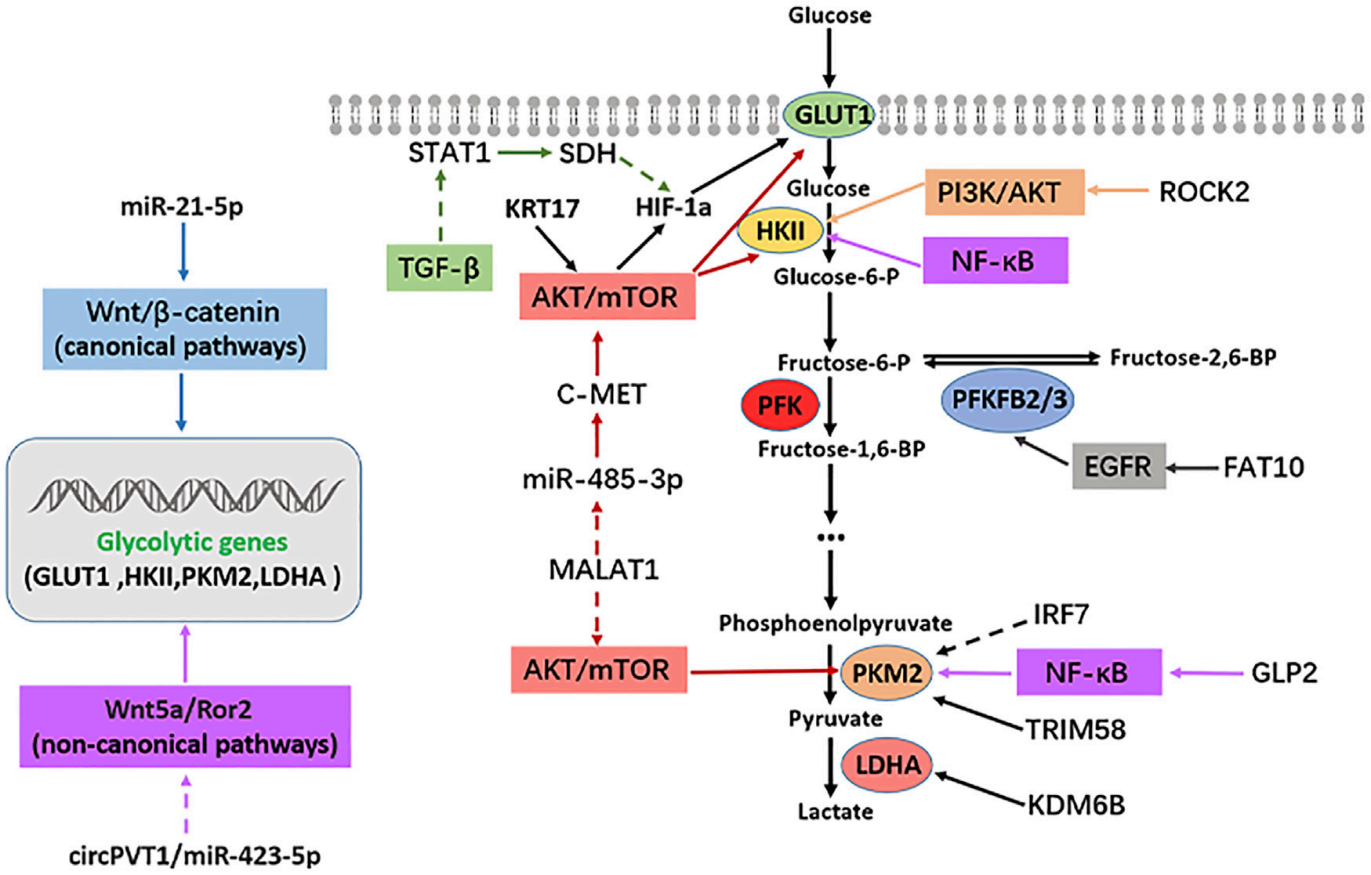
Signaling pathways regulating glycolysis in osteosarcoma. Solid arrows indicate facilitation, while dashed arrows indicate inhibition.

The rapid growth and metastasis of tumors are closely related to their enhanced glycolysis and the uptake rate. Glucose utilization efficiency in cancer cells is significantly increased (Hanahan and Weinberg, 2011; Jia et al., 2019). However, the interaction between enhanced metabolic pathways and a series of intracellular signal pathways, especially some carcinogenic signal pathways, is often an essential regulatory mechanism of tumor progression. Several signal pathways are involved in OS occurrence and development, such as PI3K/AKT, mTOR, Hippo, Notch, Wnt/β-catenin, VEGF, and TGF-β. This study summarized some classical signal pathways that participate in glycolysis and affect the progression of OS, such as PI3K/AKT, mTOR, EGFR, TGF-β, and NF-κB. The regulatory mechanism between the above signaling pathways, glycolytic enzymes, and OS is illustrated in Figure 2.

#### 2.3.1 Phosphatidylinositol-3-kinase/AKT pathway

In terms of glycolysis, PI3K can be activated by numerous proteins, which can directly activate AKT. Activated AKT can regulate the expression of glycolysis-related genes directly or indirectly (Xie et al., 2019). As a result, the PI3K/AKT pathway is thought to be very crucial in inducing glycolysis. PI3Ks is a family of lipoprotein kinases. Phosphatidylinositol (PI) catalyzes 3-hydroxy phosphorylation to produce the second messenger, which plays a role in signal transduction (Engelman, 2009). PI3Ks are divided into three types, I-III, which has a conserved kinase domain (Engelman, 2009). PI3K/AKT pathway is a carcinogenic signal pathway in many tumors, including OS. PI3K/AKT pathway has been overactivated in OS and promotes its occurrence and development of OS. Interestingly, abnormal activation of the PI3K/AKT signaling pathway is involved in various steps of pathological development of OS, such as OS occurrence, proliferation, invasion, metastasis, angiogenesis, cell cycle, apoptosis and chemotherapy resistance (Zhang et al., 2015). PI3K/AKT pathway acts on several substrate proteins, and different regulatory mechanisms are produced according to different substrate proteins. PI3K/AKT pathway is critical in regulating the glycolysis level of OS. The activated PI3K/AKT pathway positively regulates the glycolysis-related enzyme HK2 in OS. HK2 is highly expressed in OS. Inhibition of HK2 gene expression reduces OS cell proliferation and increases apoptosis, while the activated PI3K/AKT signal pathway enhances aerobic glycolysis by promoting HK2 expression (Zhuo et al., 2015). In 2017, Ru et al. (2018) found that miR-564 was expressed abnormally low in OS, and miR-564 overexpression in OS significantly suppressed the mRNA and protein expression of AKT, and attenuated OS cell glycolysis and cell proliferation. ROCK2 is highly expressed in OS tissues compared to normal tissues. The mRNA and protein expression of glycolytic enzyme HK2 is regulated by ROCK2. The specific mechanism is that ROCK2 promotes the expression of HKII by activating PI3K/AKT signal pathway, and then induces glycolysis and proliferation of OS cells (Deng et al., 2021). In summary, OS cells are maintained and developed through the PI3K/AKT pathway, which mediates glycolysis and proliferation.

#### 2.3.2 Rapamycin target protein pathway

Rapamycin target protein (mTOR) is a serine/threonine protein kinase classified as phosphatidylinositol-3-kinase (PI3K)-related protein family (Xie et al., 2019). The mTOR pathway regulates various cellular metabolism processes, such as glycolysis, amino acid metabolism, fatty acid metabolism, and lipid metabolism (Mossmann et al., 2018). Concerning glycolysis, the mTOR pathway is vital for regulating cell glycolysis. Cancer cells express glycolysis-related enzymes, which are regulated by the mTOR pathway, affecting the progression of tumors (Vijayakrishnapillai et al., 2018). The mTOR pathway has been extensively researched in tumors. The mTOR pathway is abnormally activated in many cancers, regulating the metabolism and the progression of cancer cells (Mossmann et al., 2018). In OS, the mTOR pathway is a key signal pathway that is overactivated and affects the OS progression. It is reported that multiple elements in the mTOR pathway are involved in the OS regulation (Hu K. et al., 2016). The mTOR is also often regulated by PI3K and AKT to form a PI3K/AKT/mTOR signal pathway. Interestingly, mTOR serves as an activator of AKT, so a positive feedback loop can be formed between AKT and mTOR, thus promoting the proliferation and migration of cancer cells (Zhang et al., 2015). In 2016, Wang et al. (2017) demonstrated that the mTORC1 pathway was highly activated in clinical specimens of OS. In OS, the mTORC1 regulates glycolysis, proliferation, and antioxidation of OS cells by mediating serine/glycine metabolism. Low expression of miR-485-3P is found in human OS tissues and cells with high expression of c-MET, AKT3, and MALAT1. Mechanistically, miR-485-3p binds to c-Met and AKT3 mRNAs and inhibits c-Met and AKT3/mTOR signaling pathways. Furthermore, MALAT1 interacts with miR-485-3p and inhibits c-Met and AKT3/mTOR signaling. The above regulatory mechanisms functionally inhibit glycolysis, growth, and metastasis in OS (Wang Q. et al., 2021). KRT17/AKT/mTOR/HIF-1α axis regulates glycolysis and proliferation in OS cells. KRT17 is highly expressed in OS tissues and cells, and KRT17 positively regulates the glycolysis and proliferation of OS cells. Specifically, KRT17 regulates the expression of p-AKT, p-mTOR, HIF-1a and GLUT1. The results of recovery experiment also showed that KRT17 controlled glycolysis and proliferation of OS by positively regulating AKT/mTOR/HIF-1α pathway (Yan et al., 2020). Additionally, S100A10 is highly expressed in OS tissues and cell lines, and S100 calcium-binding protein A10 (S100A10) regulates glycolytic metabolism by activating the AKT/mTOR pathway, promotes OS cell proliferation and metastasis, and inhibits apoptosis (Ling and Lu, 2022). Hence, the mTOR pathway plays a critical role in OS progression and the formulation of therapeutic strategies targeting this pathway is of utmost significance.

#### 2.3.3 Epidermal growth factor receptor pathway

Epidermal growth factor receptor (EGFR) is a member of the ErbB receptor tyrosine kinase (RTK) family, which is vital to regulating epithelial cell physiology (Lemmon et al., 2014). EGFR also regulates autophagy and metabolism (Tan et al., 2016). Xanthohumol is a prebiotic flavonoid with anticancer activity isolated from Hops (Wei et al., 2018). Xanthohumol down-regulated EGFR expression, further inhibiting the activity of AKT1 and glycolysis while promoting apoptosis in colorectal cancer (Liu W. et al., 2019). A dual novel EGFR/HER2 inhibitor, KU004, can inhibit the expression of hexokinase II (HK2) through PI3K/AKT signaling pathway, thereby inhibiting glycolysis and tumor growth (Tian et al., 2017). EGFR is often widely studied as an oncogene in tumors. In OS, an earlier study detected the expression of EGFR by immunohistochemical method. The results indicated that EGFR protein expression level was detected in 57% of all cases analyzed in the form of strong membrane staining, and six of 12 OS cells showed moderate to high expression of EGFR, which is of paramount significance for the follow-up study of EGFR in OS (Wen et al., 2007). EGFR knockdown significantly suppressed the growth and migration of OS cells. On the contrary, EGFR overexpression promotes the proliferation and metastasis of OS cells. Based on its mechanism, EGFR positively regulates the expression of p-AKT and p-ERK and promotes OS progression. Besides, the level of p-EGFR increased in OS cells treated with gemcitabine, and the inhibition of proliferation and apoptosis rate increased more significantly in OS cells treated with gemcitabine combined with EGFR knockdown (Wang S. et al., 2021b). The regulation of glycolysis by EGFR has also been reported in OS. Ubiquitin-like protein FAT10 was significantly overexpressed in OS tissues and cells. The high expression level of FAT10 was closely related to tumor growth and the short survival time of OS patients. Regarding mechanism, FAT10 directly binds to EGFR, inhibits its ubiquitination and degradation, and positively regulates the expression of glycolysis protein PFKFB3. Regarding function, FAT10/EGFR/PFKFB3 axis promotes glycolysis and proliferation of OS cells (Deng et al., 2020).

#### 2.3.4 Nuclear factor kappa B pathway

NF-κB is a family of dimer transcription factors involved in coordinating inflammatory response, innate and acquired immunity, differentiation, growth, and survival in almost all multicellular organisms (Mitchell et al., 2016). Hexokinase (HK) two is a direct transcriptional target of NF-κB. Canonical NF-κB signaling pathway inhibition reduced the expression level of HK2 in OS and inhibited OS aerobic glycolysis (Londhe et al., 2018). The activation of NF-κB leads to increased aerobic glycolysis rate and the up-regulation of GLUT3 through the loss of p53, indicating that NF-κB promotes the Warburg effect by upregulating glycolysis gene GLUT3 in cancer cells (Kawauchi et al., 2008). NF-κB can promote the Warburg effect by upregulating pyruvate kinase M2 (PKM2) (Yang et al., 2012). In OS, NF-κB inhibitors can induce apoptosis of ZOS and U2OS cells and inhibit OS’s growth. Furthermore, the authors have found that the NF-κB pathway may play a key role in OS cell survival, mainly mediated by GSK-3β and IKB kinase (IKK). When GSK-3β or IKK is blocked, IkBA is stabilized and trapped in the cytoplasm, damaging the cell survival pathway and promoting apoptosis of OS cells (Tang et al., 2012). In OS, NF-κB inhibitors can induce apoptosis of OS cells and suppress their progression. In addition, the NF-κB pathway may take part in OS cell survival, mediated by GSK-3β and IKB kinase (IKK). When GSK-3β or IKK is blocked, IkBA is stabilized and trapped in the cytoplasm, directly damaging cell survival and promoting apoptosis of OS cells (Londhe et al., 2018).

#### 2.3.5 Transforming growth factor β pathway

Transforming growth factor β (TGF-β) is the prototype of the TGF-β family of growth and differentiation factors. TGF-β is a bifunctional regulator which suppresses or promotes cell growth. TGF-β regulates numerous events in normal physiological processes, and the interference of TGF-β signals is related to the pathoge nesis of connective tissue disorder, fibrosis, and tumor (Morikawa et al., 2016). The role of the TGF-β signaling pathway in glycolysis has also been extensively studied. It was found that TGF-β1 induces PFKFB3 expression by activating p38/MAPK and PI3K/AKT signaling pathways in glioma cells, resulting in an increase in fructose 2,6-diphosphate concentration, glucose uptake, glycolysis flux, and lactic acid production (Rodriguez-Garcia et al., 2017). In lung cancer cells, TGF-β negatively regulates PDK4 expression and the downregulation of PDK4 leads to the transition from glycolysis to oxidative phosphorylation (OXPHOS) (Hua et al., 2020). It is also important to note that, TGF-β is highly expressed in OS cells and tissues. It promotes the progression of OS by activating PI3K/AKT pathway (Ma K. et al., 2020). The regulation of glycolysis by TGF-β has also been reported in OS. TGF-β weakens succinate dehydrogenase (SDH) expression by reducing the transcription factor STAT1, which leads to the accumulation of succinate and the increase of HIF-1α in OS cells, then changes the glycolysis state and drug resistance of OS cells (Xu et al., 2021). Moreover, TGF-β inhibitor RepSox inhibits OS proliferation and EMT and promotes apoptosis by inhibiting the JNK/Smad3 signal pathway (He et al., 2021).

#### 2.3.6 Wnt pathway

The Wnt signaling pathway is vital in cancer and has classical and non-classical types. It is crucial in the occurrence and development of cancer (Zhan et al., 2017). The role of the Wnt pathway in regulating glycolysis has been widely studied. In 2014, Pate et al. established that blocking Wnt signal transduction in colon cancer cells significantly reduced glycolysis metabolism and tumor growth (Pate et al., 2014). The Wnt signaling pathway was widely studied in epithelial tumors as a carcinogenic pathway. The role of the Wnt signaling pathway in mesenchymal tumors such as OS mains undefined and controversial (Singla et al., 2020). The Wnt signal pathway is involved in regulating the glycolysis of OS. Furthermore, miR-21-5p is overexpressed in OS, promoting the expression of glycolysis genes (GLUT-1, LDHA, HK2, and PKM2) by promoting Wnt/β-catenin pathway activity, thereby promoting cell proliferation and inducing apoptosis in OS (Wu Z. et al., 2021). Another study proved that the non-classical Wnt signaling pathway might also be involved in regulating glycolysis in OS. In another research, miR-423-5p was expressed in low levels in OS tissues and cells, whereas Wnt5a/Ror2 and circPVT1 were highly expressed. The circPVT1 interacts with miR-423-5p and sponges miR-423-5p, then activates Wnt5a/Ror2 signal. Functionally, circPVT1 promotes the growth and metastasis of OS through the circPVT1/miR-423-5p/Wnt5a/Ror2OS axis (Wan et al., 2021).

In summary, multiple carcinogenic pathways regulate OS glycolysis. For decades, researchers have been looking for alternatives to chemotherapy for OS, eager to find drugs with fewer side effects and higher efficacy. In OS, a considerable number of targeted signal pathway inhibitors have been shown to slow down the progression of OS significantly. The inhibitors of signaling pathways involved in glycolysis regulation in OS are summarized in Table 2.

### 2.4 Glycolysis-associated transcription factors in osteosarcoma

**FIGURE 3:**
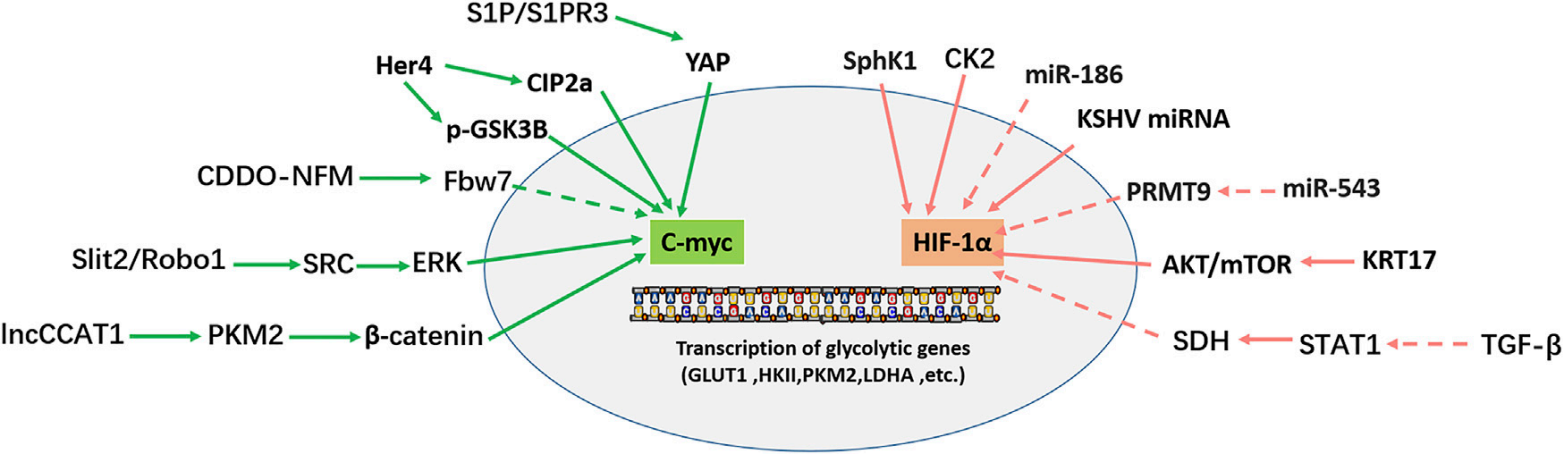
Transcription factors (C-myc, HIF-1α) regulate the transcription of glycolytic genes. Solid arrows indicate facilitation, while dashed arrows indicate inhibition.

The glycolysis pathway is regulated by a series of transcription factors, such as HIF-1α, p53, and c-myc. c-myc is linked with the cancer progression as an oncogene in most cancers and increases aerobic glycolysis by activating related genes. Under the anoxic condition, glucose metabolism affects the expression of HIF-1α, which further induces the expression of aerobic glycolysis-related enzymes. Figure 3 shows that c-myc and HIF-1α regulate glycolysis in OS.

#### 2.4.1 C-myc

The MYC family comprises three members: c-myc, N-myc, and L-myc, which are involved in various cellular activities: cell metabolism, differentiation, division, and cell death (Yoshida, 2018). In most tumors, MYC is a transcription factor; as an oncogene, MYC promotes tumor cell proliferation and metastasis. Dysfunctional MYC leads to abnormal transcription and affects cell fate by changing cell metabolism (Dang, 2016; Carroll et al., 2018; Beaulieu et al., 2020). C-myc is the most extensively studied and deregulated member of the MYC family in cancer (Duffy et al., 2021). C-myc positively regulates the glycolysis pathway *via* various mechanisms. As an important intracellular transcription factor, c-myc promotes glycolysis by transactivating and promoting the expression of genes related to glycolysis (Gruning et al., 2010; Han et al., 2012). Furthermore, as the target of several oncogenes or tumor suppressor genes, c-myc participates in regulating many signal pathways, such as MEK-ERK, Wnt/β-catenin, PI3K/AKT/mTOR, and STAT3/c-myc (Han et al., 2012; Li K. et al., 2021; Tang et al., 2022). In 2012, Han et al. (2012) found that c-myc overexpression in OS significantly enhanced the invasive ability of OS cells by promoting the MEK-ERK pathway. Moreover, c-myc is involved in cell proliferation, metastasis, apoptosis, cell cycle, and other cellular activities in OS (Wu et al., 2012; Gao J. et al., 2020; Fan et al., 2020).

C-myc is also essential for glycolysis regulation in OS. HER4 overexpression in OS enhances CIP2a-mediated c-myc S62 phosphorylation (c-myc stable) and reduces GSK3β-mediated c-myc T58 phosphorylation (c-myc unstable). Two pathways maintain c-myc stability by reducing their degradation of c-myc (Han et al., 2021). Therefore, HER4 promotes glycolysis and tumor growth in OS by upregulating c-myc positive regulation of glycolysis-related genes (GLUT1, HK2, KFPFB3, and PKM2) (Han et al., 2021). N-formyl morpholine substituent of CDDO (CDDO-NFM), a novel synthesized triterpenoid, can effectively suppress the proliferation of OS cells. CDDO-NFM promotes the degradation of c-myc by increasing the expression of Fbw7 protein, resulting in a significant decrease in the expression of glycolysis-related genes (GLUT1, PKM2, HK2, and LDHA) and blocking the glycolysis pathway (Gao et al., 2019). In 2019, Shen et al. (2019) found that S1P/S1PR3/YAP/c-myc/PGAM1 axis in OS enhances aerobic glycolysis. Sphingosine 1-phosphate receptor 3 (S1PR3) is a member of the G protein-coupled receptor (GPCRs) family and is critical in tumor imaging and targeted therapy. S1PR3 and its specific ligand S1P are highly expressed in OS. S1P/S1PR3 inhibits the phosphorylation of YAP and promotes the entry of YAP into the nucleus. YAP entering the nucleus forms a YAP/c-myc complex with c-myc. C-myc enhances glycolysis by promoting the transcription of the glycolytic enzyme PGAM1. TY52156 (S1PR3 antagonist) and methotrexate showed a synergistic effect on inhibiting OS cell growth *in vitro* and *in vivo* (Shen et al., 2019). Zhao et al. (2018) found that the high expression of slit guidance ligand-2 (SLIT2) and roundabout guidance receptor-1 (ROBO1) in OS is closely related to the decrease in overall survival rate in OS patients. SLIT2/ROBO1 axis enhances the expression of glycolysis-related genes by activating SRC/ERK/c-myc/PFKFB2 signal pathway. Regarding function, the SLIT2/ROBO1 axis promotes glycolysis and proliferation of OS cells, inhibits apoptosis, and promotes OS. The lncRNA colon cancer-associated transcript-1 (lncCCAT1) interacts with PKM2. PKM2 further promotes the phosphorylation of sterol regulatory element-binding protein 2 (SREBP2) and activates MYC transcriptional activity, thereby promoting glycolysis and adipogenesis. In addition, CDC25A dephosphorylates PKM2 and promotes the transactivation of PKM2, which in turn enhances the transcriptional expression of c-myc and upregulates the expression of glycolysis genes (PKM2, LDHA and GLUT1). The above mechanisms promote the Warburg effect, adipogenesis, and OS cell growth (Pu et al., 2022).

#### 2.4.2 HIF-1α

Hypoxia-inducible factor-1α (HIF-1α) is a significant transcription factor that improves cell viability in a hypoxic environment, which is precisely regulated by hypoxia and hyperglycemia. HIF-1α and glucose interact with each other. HIF-1α induces the expression of glycolytic enzymes, while glucose metabolism affects the accumulation of HIF-1α in some cells (Xiao et al., 2013). In addition, HIF-1α is one of the critical transcription factors in tumor progression, and cancer targeted therapy. The effect of HIF-1α varies with or without oxygen. In an oxygen immersion environment, HIF-1α is completely inactivated and destroyed by the ubiquitin-proteasome pathway (UPP). However, in an anaerobic environment, it escapes damage, enters the nucleus, and then upregulates many genes involved in cancer progression (Rashid et al., 2021). HIF-1α activates the transcription of numerous genes related to tumor biology, including apoptosis, cell proliferation, angiogenesis, and glucose metabolism. Hypoxia and genetic changes in tumors can lead to HIF-1α overexpression. Compared with normal tissues, HIF-1α is highly expressed in different kinds of tumors, such as colon cancer, gastric cancer, lung cancer, skin cancer, pancreatic cancer, and breast cancer, including human OS, which is significantly associated with metastasis (Zhong et al., 1999). HIF-1α is strongly expressed in OS tissues compared with para cancerous tissues, and is associated with poor prognosis in patients with OS (Yang et al., 2007). In 2015, Guan et al. suggested that hypoxia promotes the metastasis of OS cells by activating HIF-1α/CXCR4 pathway (Guan et al., 2015). Furthermore, hypoxia and increased HIF-1α protein expression regulate the growth and metastasis of OS cells by upregulating VEGF expression (Liu M. et al., 2016). Simultaneously, HIF-1α overexpression upregulated the level of NDUFA4L2, thus promoting the metastasis and epithelial-mesenchymal transformation of OS cells by inhibiting ROS production (Xu et al., 2020). Yan et al. (2020) reported that KRT17 is highly expressed in osteosarcoma tissues and osteosarcoma cell lines, and promotes the proliferation and glycolysis of osteosarcoma cells by activating the AKT/mTOR/HIF1α pathway. TGF-β negative transcription factor STAT1 further inhibited the expression of succinate dehydrogenase (SDH). The decrease of SDH leads to HIF-1α upregulation, which changes the metabolic level of glucose and the drug resistance of OS cells (Xu et al., 2021). The miR-186 inhibits the proliferation, invasion, and glycolysis of OS cells by targeting HIF-1α and pituitary tumor transforming gene 1 (PTTG1) (Xiao et al., 2018). Interestingly, there is a negative correlation between the expression of miR-543 and protein arginine methyltransferase 9 (PRMT9) in OS samples and cell lines. There is a positive regulation of glycolysis by the miR-543/PRMT9/HIF-1α axis, thus promoting OS progression (Zhang H. et al., 2017a). Additionally, in the OS U2OS cell line, a Ser/Thr protein kinase CK2, which is highly expressed in many cancers, enhances glycolysis by upregulating HIF-1α expression (Zonta et al., 2021). Another study found that sphingosine kinase 1 (SphK1) increases the glycolysis level of OS cells by over-regulating HIF-1α expression and participates in the progression of doxorubicin resistance in OS cells (Ren and Su, 2020). Furthermore, Kaposi’s sarcoma herpesvirus (KSHV) miRNA promotes HIF-1α expression in OS to enhance glycolysis (Yogev et al., 2014).

### 2.5 Glycolysis-associated non-coding RNAs in osteosarcoma

Transcriptional regulation in mammalian cells is a complex regulatory process vital for cell life activities, such as differentiation, development, and metabolism (Carninci et al., 2005; Gustincich et al., 2006). There are numerous types of non-coding RNAs (ncRNAs), such as miRNAs, piRNAs, snoRNAs, lncRNAs and circRNA (Esteller, 2011). Some of these ncRNAs, such as miRNA, lncRNA and circRNA, have been researched extensively. Some scholars believe these ncRNAs are epigenetic regulators that affect various biological processes, including bone metabolism (Yang et al., 2020). This study reviewed the effects of three kinds of miRNA (Table 3), lncRNA (Table 4), and circRNA (Table 5) on glucose metabolism in OS cells. The ncRNAs can directly or indirectly regulate the expression of glycolysis-related enzymes to affect cell glycolysis and then affect OS proliferation, invasion, migration, and other malignant behavior.

**TABLE 3:** miRNAs regulate glycolysis enzymes in osteosarcoma.

miRNAs	Target glycolysis enzymes	Involvement of factors	Function	References
miR-34c-5p	ALDOA	miR-34c-5p/ALDOA	Down-regulate	Shen et al. (2020)
miR-132	Glut1	miR-132/Glut1	Down-regulate	Rafat et al. (2021)
miR-150	GLUT1	miR-150/GLUT1	Down-regulate	Yuan et al. (2017)
miR-522-3p	GLUT1	miR-522-3p/GLUT1	Up-regulate	Rong Chen et al. (2019)
miR-328-3p	GLUT1	miR-328-3p/LAT1/SLC7A5/GLUT1	Down-regulate	Yi et al. (2020)
miR-485-3p	GLUT1, HK2, PKM2	c-MET, AKT3/mTOR/GLUT1	Down-regulate	Qing Wang et al. (2021)
miR-21-5p	GLUT1, LDHA, HK2, PKM2	miR-21-5p/Wnt/β-catenin/GLUT	Up-regulate	Ziyan Wu et al. (2021)
miR-186	HIF-1α	miR-186/HIF-1	Down-regulate	Xiao et al. (2018)
miR-543	HIF-1α	miR-543/PRMT9/HIF-1α	Up-regulate	Heng Zhang et al. (2017a)
miR-1225-3p	HK2	miR-1225-3p/KCNH1	Down-regulate	Zhengmao Li et al. (2021)
miR-125b	HK2	miR-125b/HK2	Down-regulate	Wu et al. (2017)
miR-134	HK2	ELK1/miR-134/PTBP1	Down-regulate	Zhang et al. (2021b)
miR-143	HK2	lncRNA-SARCC/miR-143/HK2	Down-regulate	Wen et al. (2020)
miR-144	HK2	miR-144/CXCR4	Down-regulate	Jun Liu et al. (2020b)
miR-181b	HK2	miR-181b/HK2	Down-regulate	Gu et al. (2020)
miR-335-5p	HK2, PKM2	miR-335-5p/Myh9/HK2, PKM2	Down-regulate	Cao et al. (2021)
miR-372-3p	HK2	miR-372-3p/MAPK7/HK2	Down-regulate	Yuanpeng Gao et al. (2020)
miR-423-5p	HK2, PKM2, GLUT1, LDHA	miR-423-5p-Wnt5a/Ror2/HK2	Down-regulate	Wan et al. (2021)
miR-615	HK2	PI3K/AKT/HK2	Down-regulate	Limin Sun et al. (2020)
miR1244	HK2, LDHA	miR1244/TRIM44	Down-regulate	Huo and Dou, (2021)
miR-497	HK2	miR-497/HK2	Down-regulate	Song et al. (2017)
miR-185	HK2	miR-185/HK2	Down-regulate	Chaojian Liu et al. (2019)
miR-323a-3p	LDHA	miR-323a-3p/LDHA	Down-regulate	Hanwen Chen et al. (2018)
miR-329-3p	LDHA	miR-329-3p/LDHA	Down-regulate	Gang Li et al. (2021)
miR-33b	LDHA	miR-33b/LDHA	Down-regulate	Zheng et al. (2018)
miR-409-3p	LDHA	miR-409-3p/LDHA	Down-regulate	Zhang et al. (2021a)
miR-15b-5p	PDK4	miR-15b-5p/PDK4	Down-regulate	Weng et al. (2018)
miR-1297	PFKFB2	miR-1297/PFKFB2	Down-regulate	Pan et al. (2020)
miR-26b	PFKFB3	miR-26b, PFKFB3	Down-regulate	Zheng et al. (2015)
miR-26b	PFKFB3, LDHA, GLUT-1	miR-26b/PFKFB3	Down-regulate	Du et al. (2015)
miR-542-3p	PFKM	miR-542-3p/PFKM	Down-regulate	Jia et al. (2021)
miR-23b-3p	PGC1α	miR-23b-3p/PGC1α	Down-regulate	Zhu et al. (2019)
miR-365a-3p	PGK1	miR-365a-3p/PGK1	Down-regulate	Pan et al. (2022)
miR-1294	PKM2	miR-1294/PKM2	Down-regulate	Yuan et al. (2020)
miR-491-5p	PKM2	miR-491-5p/PKM2	Down-regulate	Ting Chen et al. (2018)
miR-198	PFKFB4	miR-198/E2F2	Down-regulate	Liu et al. (2022)
miR-578	LDHA, PDK1	miR-578-LDHA/PDK1	Down-regulate	Hu et al. (2021)

**TABLE 4:** lncRNAs regulate glycolysis enzymes in osteosarcoma.

lncRNA	Target glycolysis enzymes	Involvement of factors	Function	References
lncRNA HAND2-AS1	GLUT1	lncRNA HAND2-AS1/GLUT1	Down-regulate	Shunguang Chen et al. (2019)
lncRNA PVT1	HK2	lncRNA PVT1/miR-497/HK2	Up-regulate	Song et al. (2017)
lncRNA FEZF1-AS1	HK2	lncRNA FEZF1-AS1/miR-144/CXCR4	Up-regulate	Jun Liu et al. (2020b)
lncRNA SARCC	HK2	lncRNA SARCC/miR-143/HK2	Down-regulate	Wen et al. (2020)
lncRNA TUG1	HK2	lncRNA TUG1/HK2	Up-regulate	Han et al. (2018)
lncRNA CCAT1	PKM2	lncRNA CCAT1/PKM2/SREBP2	Up-regulate	Pu et al. (2022)
lncRNA HCG18	PGK1	lncRNA HCG18/miR-365a-3p/PGK1	Up-regulate	Pu et al. (2022)
lncRNA XLOC_005950	PFKM	lncRNA XLOC_005950/miR-542-3p/PFKM	Up-regulate	Jia et al. (2021)
lncRNA KCNQ1OT1	ALDOA	lncRNA KCNQ1OT1/miR-34c-5p/ALDOA	Up-regulate	Shen et al. (2020)

**TABLE 5:** cicrRNAs regulate glycolysis enzymes in osteosarcoma.

circRNA	Target glycolysis enzymes	Involvement of factors	Function	References
CircRNA PVT1	HK2, PKM2, GLUT1, LDHA	CircRNA PVT1/miR-423-5p/Wnt5a/Ror2/HK2	Up-regulate	Wan et al. (2021)
CircRNA FAT1(e2)	HK2	CircRNA FAT1(e2)/miR-181b/HK2	Up-regulate	Gu et al. (2020)
CircRNA 0056285	HK2, LDHA	CircRNA 0056285/miR-1244/TRIM44/HK2	Up-regulate	Huo and Dou, (2021)
CircRNA 0016347	HK2	CircRNA 0016347/miR-1225-3p/KCNH1/HK2	Up-regulate	Zhengmao Li et al. (2021)
CircRNA Circ_0001721	HK2	CircRNA Circ_0001721/miR-372-3p/MAPK7/HK2	Up-regulate	Yuanpeng Gao et al. (2020)
CircRNA CNST	LDHA, PDK1	CircRNA CNSTmiR-578/LDHA, PDK1	Up-regulate	Hu et al. (2021)
CircRNA ATRNL1	LDHA	CircRNA ATRNL1/miR-409-3p/LDHA	Up-regulate	Zhang et al. (2021a)
CircRNA 1244	HK2, LDHA	CircRNA 1244/miR1244/HK2, LDHA	Up-regulate	Huo and Dou, (2021)
CircRNA TP2A2	HK2, PKM2	CircRNA TP2A2/miR-335-5p/Myh9/HK2, PKM2	Up-regulate	Cao et al. (2021)

#### 2.5.1 miRNAs

miRNA is a highly conserved endogenous non-protein-coding RNA about 19–25 nucleotides in length, whose transcription process is independent of other genes. miRNAs degrade or induce translational silencing by binding to the 3′ untranslated regions (3′-UTRs) of target mRNAs, affecting cell proliferation, differentiation, apoptosis, and ontogeny (Jackson and Standart, 2007). As revealed in Table 3, most miRNAs inhibit the glycolysis and progression of OS cells. Chen et al. reported that miR-522-3p and GLUT1 are highly expressed and positively correlated with OS and that high levels of miR-522-3p are associated with low survival rates. Regarding the mechanism, miR-522-3p increases GLUT1 expression and glucose uptake, thus promoting the proliferation of OS cells (Chen R. et al., 2019). In addition, miR-543 is highly expressed in OS tissues and cells and positively regulates glycolysis through miR-543/PRMT9/HIF-1α axis to promote OS progression (Zhang H. et al., 2017a). miR-328-3p regulates GLUT1-mediated glucose uptake and metabolism in OS cells, and in addition, hBERA/miR-328 combined treatment with cisplatin or doxorubicin demonstrated strong synergy in inhibiting OS cell proliferation (Yi et al., 2020). Also, miRNAs can regulate glycolysis in OS cells by acting on signaling pathways. This study demonstrated that miR-485-3p was decreased and c-MET, AKT3, and MALAT1 were increased in human OS tissues and cells. miR-485-3p directly binds to c-MET and AKT3 mRNA and inhibits OS cell glycolysis, proliferation, migration, and invasion by reducing glycolysis-related proteins and migration-related proteins by inhibiting c-MET and AKT3/mTOR pathways (Wang Q. et al., 2021). Whereas miR-21-5p is overexpressed in OS. miR-21-5p upregulates Warburg effector proteins (GLUT1, LDHA, HK2, and PKM2) by activating Wnt/β-catenin signaling, promoting OS cell proliferation and metastasis (Wu Z. et al., 2021).

#### 2.5.2 lncRNAs

LncRNAs are non-coding RNA with a length of more than 200 nucleotides, but due to the lack of open reading frames, they cannot encode proteins (Prensner and Chinnaiyan, 2011). However, recent studies have stated that lncRNAs not only directly regulate gene expression but also interact with miRNAs as competing endogenous RNAs (Zheng et al., 2019). As illustrated in Table 4, most lncRNAs positively regulate glycolysis, while a few lncRNAs inhibit the glycolysis pathway of OS cells. For example, lncRNAHAND2-AS1 inhibits glycolysis through negative regulation of GLUT1 (Chen S. et al., 2019). And lncRNA SARCC inhibits glycolysis through negative regulation of HK2 (Wen et al., 2020). In addition, lncRNA SNHG4 is highly expressed in OS tissues and cell lines, miR-377-3p is downregulated in OS, and SNHG4 acts as a competing endogenous RNA with sponge miR-377-3p to promote OS tumor proliferation and migration (Huang Y. F. et al., 2020). LncRNA FTX expression has also upregulated in OS, and lncRNA FTX inhibition suppressed the proliferation and migration of OS cells by regulating miR-320a/TXNRD1 (Huang S. et al., 2020). Similarly, lncRNA ROR1-AS1 is upregulated in OS tissues, ROR1-AS1 acts as a sponge for miR-504, and ROR1-AS1 overexpression suppressed miR-504 expression in miR-63 cells. LncRNA ROR1-AS1 accelerates OS invasion and proliferation by regulating miR-504 (Wu et al., 2020). LncRNAs can also regulate the proliferation or apoptosis of OS cells through signaling pathways. For instance, lncRNA MEG3 overexpression in OS cells can inhibit their proliferation and promote their apoptosis by inhibiting the Notch signaling pathway (Chen et al., 2020). Furthermore, lncRNA GAS5 is downregulated in human OS tissues and cell lines, and GAS5 acts as a competing endogenous RNA regulating the PI3K/AKT pathway by sponging miR-23a-3p, promoting PTEN expression and inhibiting PTEN expression in OS cell growth and invasion (Liu J. et al., 2020a).

#### 2.5.3 Circular RNAs

Circular RNAs (circRNAs) are endogenous non-coding RNAs widely present in eukaryotic cells. Recently, circRNAs have been demonstrated to act as competing endogenous RNAs, namely miRNA sponges, to regulate the expression of target genes, as well as act as transcriptional regulators or RNA-binding proteins to indirectly regulate genes at the post-transcriptional level (Jeck et al., 2013; Du et al., 2016). CircRNAs have also been reported to promote OS glycolysis, as illustrated in Table 5. For example, circTADA2A is highly expressed in OS tissues and cell lines, and circTADA2A can easily sponge miR-203a-3p to upregulate CREB3 expression, which was identified as a driver gene in OS that promotes progression and metastasis (Wu et al., 2019) circCAMSAP1, also known as hsa_circ_0004338, is significantly upregulated in human OS tissues and cell lines and promotes OS progression and metastasis by sponging miR-145-5p and regulating FLI1 expression (Chen Z. et al., 2021). In addition, OS circ_001422 expression was significantly increased in cell lines and tissues, and the competitive interaction between circ_001422 and miR-195-5p increased FGF2 expression and initiated PI3K/Akt signaling, i.e., circ_001422 *via* regulation of miR-195-5p/FGF2/PI3K/Akt axis accelerates OS tumorigenesis and metastasis (Yang et al., 2021). Similarly, circRNA_103801 can accelerate the proliferation of OS cells by sponging miR-338-3p and regulating HIF-1/Rap1/PI3K-Akt pathway (Li Z. Q. et al., 2021).

## 3 Conclusion and future perspectives

**FIGURE 4:**
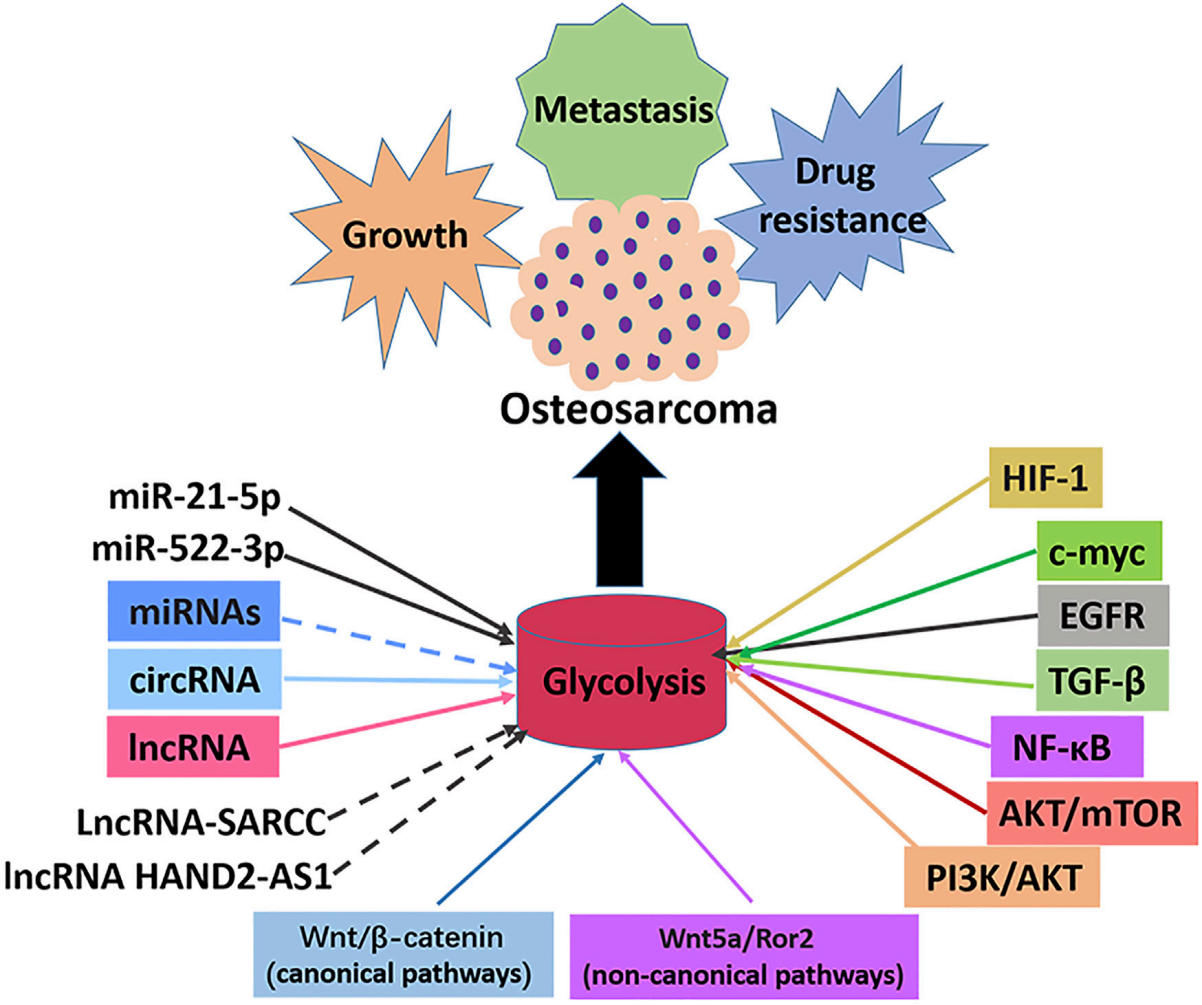
Signal pathways, transcription factors and ncRNAs that regulate glycolysis in osteosarcoma. The enhancement of glycolysis in osteosarcoma promotes the malignant behaviors such as proliferation, metastasis and drug resistance of osteosarcoma cells. Most miRNAs inhibit the progression of osteosarcoma by inhibiting glycolysis, with the exception of miR-21-5p and miR-522-3p. Most lncRNAs promotes the progression of osteosarcoma by promoting glycolysis, with the exception of lncRNA-SARCC and lncRNAHAND2-AS1. All circRNAs promote osteosarcoma progression by promoting glycolysis. Transcription factors c-myc and HIF-1α promote glycolysis and osteosarcoma progression. Signal pathways PI3K/AKT, AKT/mTOR, NF-κB, TGF-β, EGFR, Wnt/β-catenin, Wnt5a/Ror2 promote glycolysis pathway and osteosarcoma progression.

The abnormal activity of glycolysis is a sign of metabolic reprogramming of cancer cells and is very important for developing tumors. Similarly, hyperactive glycolysis in OS, a highly invasive tumor, maintains and promotes various malignant behaviors of OS cells. A series of enzymes mainly catalyzes the glycolysis pathway to promote the transformation of energy substances. In cancer cells, oncogenes and tumor suppressor genes change the level of glycolysis by directly or indirectly regulating the expression and enzyme activity of glycolysis-related genes. Numerous studies have shown that the classical signaling and glycolysis pathways in OS overlap and influence each other. This study reviewed several key enzymes involved in glycolysis, classical and non-classical signal pathways involved in glycolysis, and the role of intracellular transcription factors in the progression and treatment of OS (Figure 4). As a result of the complexity of the glycolysis pathway in OS, no single inhibitor of glycolysis can be effective in treating OS. It is imperative to find more key genes in the glycolysis pathway and OS occurrence and development to help find more effective metabolic drug targets. A summary of some inhibitors involved in the glycolysis pathway of OS is presented in this article. There is no reliable evidence that these inhibitors inhibit cellular glycolysis. However, the combined use of signal pathways targeting small molecules and glycolysis targeted inhibitors provides new insights for treating OS. Since most current research results were from OS cell experiments, the real prospect of clinical application remains unknown, so developing glycolysis therapy for OS is a challenge. In conclusion, the co-development of the glycolysis pathway, conducting basic research on OS progression, and providing corresponding clinical treatment will benefit OS patients.
